# Advancing school dropout early warning systems: the IAFREE relational model for identifying at-risk students

**DOI:** 10.3389/fpsyg.2023.1189283

**Published:** 2023-07-31

**Authors:** Angelina Nunes de Vasconcelos, Leogildo Alves Freires, Gleidson Diego Lopes Loureto, Gabriel Fortes, Júlio Cezar Albuquerque da Costa, Luan Filipy Freire Torres, Ig Ibert Bittencourt, Thiago Damasceno Cordeiro, Seiji Isotani

**Affiliations:** ^1^Institute of Psychology, Federal University of Alagoas, Maceió, Brazil; ^2^Center of Excellence for Social Technologies (NEES), Federal University of Alagoas, Maceió, Brazil; ^3^Education Center, Federal University of Roraima, Boa Vista, Brazil; ^4^Department of Psychology, Alberto Hurtado University, Santiago, Metropolitan Region (RM), Chile; ^5^Computing Institute, Federal University of Alagoas, Maceió, Brazil; ^6^Institute of Mathematics and Computer Science, University of São Paulo, São Carlos, Brazil

**Keywords:** school dropout, early warning system, large scale analysis, school dropout factors, school dropout prevention

## Abstract

**Introduction:**

There is a global effort to address the school dropout phenomenon. The urgency to act on it comes from the harmful evidence that school dropout has on societal and individual levels. Early Warning Systems (EWS) for school dropout at-risk student identification have been developed to anticipate and help schools have a better chance of acting on it. However, several studies point to a doubt that Correct EWS may come too late because they use only publicly available and general student and school information. We hypothesize that having a tool to assess more subjective and inter-relational factors would help anticipate where and when to act to prevent school dropout. This study aimed to develop a multidimensional measure for assessing relational factors for predicting school dropout (SD) risk in the Brazilian context.

**Methods:**

We performed several procedures, including (a) the specialized literature review, (b) the item development of the Relational Factors for the Risk of School Dropout Scale (IAFREE in Portuguese), (c) the content validity analysis, (d) a pilot study, and (e) the administration of the IAFREE to a large Brazilian sample of high school and middle school students (N = 15,924).

**Results:**

After the theoretical steps, we found content validity for five relational dimensions for SD (Student-School, Student-School Professionals, Student-Family, Student-Community, and Student–Student) that include 12 facets of risk factors. At the empirical stage, confirmatory analysis corroborated the proposed theoretical model with 12 first-order risk factors and 5 s-order dimensions (36 items). Further, through the Item Response Theory analysis, we assessed the individual item parameters of the items, providing a brief measure without losing psychometric quality (IAFREE-12).

**Discussion:**

We discuss how this model may fill gaps in Correct EWS models and how to advance it. The IAFREE is a good measure for scholars investigating the risk of SD. These results are important for implementing an early warning system for SD that looks into the complexity of the school dropout phenomenon.

## 1. Introduction

Despite the well-documented evidence showing the adverse life outcomes associated with dropping out of school, only recently, the phenomenon of school dropout has been conceived as a worldwide public health issue (e.g., Centers for Disease Control and Prevention; American Public Health Association, 2018). Although the definition of school dropout has been under discussion for a long time (Gutiérrez-de-Rozas et al., 2022) as this work was conducted in Brazil and the Early Warning System developed here is aimed at this context, we will use Brazilian governmental understanding of school dropout, which is the absence or early leaving school for, at least, one year (Brasil Instituto Nacional de Estudos e Pesquisas Educacionais Anísio Teixeira (INEP), 2021). Using this simple definition help us in concentering our efforts in identifying students in the process of dropping out and not just identify risk factors that impacted the decision of leaving the school system.

School dropout, in this sense, is both a public health issue and an economic concern (Wood et al., 2017). It is an economic concern because dropping out of school is directly associated with high rates of unemployment in the future (Marchbanks et al., 2014; Ecker-Lyster and Niileksela, 2016; Vinas-Forcade et al., 2021), but other consequences of this phenomenon are related to mental health and social issues. Previous research has shown that early school leaving predicts high levels of stress, mental disorders, decreased quality of life, illiteracy, and increased crime and poverty (De Witte et al., 2013; Dever and Raines, 2013; Nelson et al., 2016; Vinas-Forcade et al., 2021). Therefore, there is an urgency to support students and reduce school dropout rates worldwide (Barfield et al., 2012). A significant alternative being developed worldwide is the creation of Early Warning Systems (EWS) for identifying at-risk school dropout students and helping stakeholders to come up with solutions for this phenomenon (Faria et al., 2018).

Empirical studies have sought to identify factors associated with dropout to shed light on detecting students at risk of dropping out, planning interventions, and public policy related to solving the graduation problem (Bowers and Sprott, 2012). For example, some studies pointed out that the students at risk for dropping out of school are associated with four essential conditions: failing core academic courses, excessive absenteeism, failure to be promoted to the next grade level, and being detached in the classroom (Neild and Balfanz, 2006). Furthermore, other evidence suggests that dropping out of school can be motivated by ongoing failure, retention, school policies, and being of age to finally leave the system (Bradshaw et al., 2008; Bowers, 2010).

Another body of evidence has sought to identify student- and school-level factors for school dropout. For example, Wood et al. (2017) found significant student-level predictors in the United States, such as academic achievement, school retention, sex, family socioeconomic status (SES), and extracurricular involvement. For the school level, predictors were school SES and school size. Hirakawa and Taniguchi (2021) observed that in rural Cambodia, school dropout rates at the student level are predicted by age at first school entry, gender, relative achievement in class, parental educational attainment, economic status, p, educational aspiration, teacher interaction and living with parents. Another salient factor related to school dropout was time spent helping the family with household chores. At the school level, teacher absence, meaningful interaction with teachers in higher-grade, and mean test achievement in the lower grades were associated with dropout (Hirakawa and Taniguchi, 2021).

In addition, some protective factors for early school leaving were addressed in the literature. For example, Lansford et al. (2016) observed that being in a romantic relationship for a more extended period, having an excellent mother–child relationship and self-reported father–child relationship quality during early adulthood, and strong affiliating with a peer group during early adulthood are associated with lower rates of school drop addition, pout. In addition, Hirakawa and Taniguchi (2021) found that reducing teacher absence and late school entry were protective against dropping out of school. Moreover, the literature has reported that engagement in extracurricular activities (e.g., Mahoney and Cairns, 1997; Bowers et al., 2012) and a more supportive relationship with parents and teachers (Pedditzi et al., 2022) have been identified as protective factors against dropout.

Before planning any interventions for the school dropout issue, the first step consists of its quantification. Identifying and quantifying variables associated with school dropout is a vital issue in developing a nationwide type of EWS and, therefore, why it is a pressing issue to come up with assessment tools that are more and more enabling of acting as soon as possible to avoid school dropout. According to the most recent statistics on trends at the global and regional levels (a revised calculation method that provides more precise estimates of the out-of-school population; UNESCO Institute for Statistics (UIS), 2019), about 258 million children and youth were out of school ending in 2018 (e.g., 59 million children of primary school age, 62 millions of lower secondary school age, and 138 millions of upper secondary school age). Concerning out-of-school rates and numbers (millions) by region, the following data were observed: Sub-Saharan Africa (97.5), Southern Asia (93.0), Eastern and South-Eastern Asia (32.6), Central Asia (1.1), Northern Africa and Western Asia (17.1), Europe and Northern America (4.4), and Oceania (0.7). The number observed for Latin America and the Caribbean was 12.0 (UNESCO Institute for Statistics (UIS), 2019).

Then, the school dropout statistics are essential for estimating the educational system’s performance, appraising policies, and developing new strategies to improve achievements (Allensworth and Clark, 2020). In response to the limitations of a non-existing tool for the evaluation of risk factors for school dropouts in Brazil, this study aimed to conceptually discuss what type of information is necessary to advance EWS capacity to act sooner and develop an assessment tool that uses relational factors for predicting school dropout (SD) risk: the Relational Factors for the Risk of School Dropout Scale (IAFREE). Further, we present the procedures describing (a) the specialized literature review, (b) the development of the Relational Factors for the Risk of School Dropout Scale (IAFREE), (c) the content validity analysis, (d) a pilot study, and (e) the administration of the IAFREE to a sizeable Brazilian sample of high school and middle school students.

## 2. Advancing the theoretical model of early warning systems (EWS)

Several theoretical, empirical, and evidence-informed models are explicit or implicit in constructing an EWS. We present and discuss what has been considered the most relevant models both from a scientific scope and a public relevance one (Frazelle and Barton, 2006; UNICEF, 2014, 2018a,b).

First, we have the most known school dropout conceptualization and prediction model. Before Early Warning Systems, models with the ABC (Wilhoit and Roesch, 1989; Christenson, 1995) focused on changing students’ behavior to increase school attendance. However, only in early 2000 a model to be implemented nationwide and based on an acronym comprised of three core components—Attendance (A), Behavior (B), and Course Performance (C) is created (Civic Enterprises and Everyone Graduates Center, 2011). This model is based on the idea that student absence should not be lower than 20 days (or 10% of classes) in a year, no more than two episodes of mild or severe misbehavior infraction, and the capacity to achieve basic skills for each grade throughout their academic trajectory. The ABC model is designed to assist schools in identifying students who may be in danger of dropping out by closely monitoring these three decisive factors. Specifically, school staff must pay attention to declining student attendance, in which case they might contact the student’s family, offer transportation assistance, or provide incentives for attending school. They must monitor student behavior, from disciplinary incidents to negative interactions with peers and staff, to determine which students need additional assistance, such as counseling or mentoring. Lastly, for the ABC model to be successful, staff must closely observe student course performance, including grades and test scores, to signal when a student may be in academic jeopardy. In this case, targeted support like tutoring or educational interventions should be deployed. The ABC model provides a simple yet effective early alert system for recognizing students at risk of dropping out. It subsequently provides the targeted support needed to empower them to stay on track (Balfanz and Byrnes, 2019). As Frazelle and Nagel (2015) points, EWS benefit from having reduced and simpler versions of data process to enhance government action, however, more data can be added to this model to a better understanding of school dropout factors of risk and protection.

The UNESCO model represents a framework of five key factors contributing to school dropout, including individual, family, school, community, and systemic components. This model promotes the idea that schools should evaluate and monitor these factors, utilizing data analysis to identify students at risk of dropping out. Schools are then encouraged to provide targeted support through counseling, mentoring, tutoring, and extracurricular activities and involve relevant stakeholders in the mission to prevent the issue of dropouts. The UNESCO model has been implemented in many countries, though adapted to the local context, while always honoring the underlying principles of early identification and targeted support. Ultimately, this model is an effective early alert system for school dropouts and can help save students and communities.

While the two models support their cause, their focus and approach differ significantly. Whereas the ABC model focuses on specific variables to assess and predict school dropout, the UNESCO model promotes early identification and gives support to stakeholders to create a more significant impact by promoting children’s rights and well-being. What is missing, then, is a more contextualized and personalized assessment of students’ variables in relation to critical factors of their education life. In this sense, the simplicity and intuitiveness of both models may also make invisible other processes that can account for school dropout risks, such as familial relationships, peer relations, and school climate. Thus, to progress the index of real-time prevention against school dropout, we propose the adoption of an instrument that can access personalized and localized information from both students and school staff to predict better, not only identify the at-risk group of students.

### 2.1. From multidimensional to relational early warning systems

Early warning systems are used to identify students at risk of dropping out. School dropout early warning systems are designed to identify students at risk of leaving school before completing their education. These systems rely on various data sources, including academic performance, attendance records, and socio-demographic information, to identify students who may be struggling and require additional support. Early warning systems use statistical and machine learning techniques to analyze student data and identify patterns indicative of dropout risk. Heppen and Therriault’s (2008) research has shown that early warning systems based on structured data, such as academic performance, effectively predict a student’s likelihood of dropping out. For example, in Wisconsin, the Dropout Early Warning System (DEWS) is already in place and can predict the graduation rate of over 225,000 students (Knowles, 2015). Using a data mining approach, its proponents may predict students at risk. Through early warning systems, it is possible to create proactive measures to prevent students from dropping out of school. However, it seems that this approach relies on end-points of a school trajectory (such as bad grades), and we propose that we should start looking for variable relations that can help predict and act as soon as possible.

Early warning systems can consider these factors to provide a more comprehensive picture of a student’s risk of dropping out, such as social and economic status, academic performance, and family background (Rumberger and Lim, 2008). In a study conducted by Braz et al. (2019), it was discovered that an Early Warning System (EWS) has the potential to accurately predict when a student is at risk of disengaging from their academic pursuits. Once this disengagement has been detected, the system is designed to notify teachers, who can intervene promptly and effectively. By utilizing an EWS, teachers, and administrators can stay informed about struggling students, allowing them to provide tailored support and resources to help these students stay on track and achieve their academic goals. This proactive approach to addressing student disengagement can ultimately lead to improved educational outcomes and increased academic success rates for at-risk students.

A successful example is Chile’s Sistema de Alerta Temprana (Early Alert System), which follows the UNESCO model of school dropout guidelines. This system utilizes a trifecta of data on attendance, academic performance, and behavior (which is closely related to the ABC model) to identify students who are at risk of dropping out. Doing so gives these students the necessary targeted support from a team of academic mentors, counselors, and other experts to ensure their educational well-being. An advantage of this system is the accessibility of the admissions data, spoken of by the school principalities and other interested parties, which allows them to identify and intervene with cases as soon as they appear.

Ultimately, early alert systems aim to prevent school dropouts and promote an environment where academic excellence is within reach of every student. However, this strategy often results in identifying students already in the process of school dropout (not before it happens) and hinders the ability to accurately predict and detect at-risk students in time to provide interceding support. By identifying students who are at risk of dropping out early, school dropout early warning systems enable educators to develop proactive measures to prevent students from leaving school prematurely. These measures may include academic interventions, counseling services, and other forms of support to help students stay on track and complete their education.

Historically, punitive, and guilt-inducing approaches to understanding and combating dropout have been adopted in Brazil, for example, punishing students and their families for excessive absences and accusing them of “disinterest” in school (Gil, 2018). In opposition, a more comprehensive approach that involved the socio-educational monitoring of students emerged. This model considered that school dropout was not just an individual problem, but was often related to social, economic, and family factors. Thus, social and psychosocial assistance programs emerged that aimed to identify and address the underlying causes of dropout. Recognizing the importance of family and community context in dropout, approaches involving partnership between school, family, and community emerged (Samuel and Burger, 2020; Piscitello et al., 2022). The creation of links between these actors, the strengthening of the school-family relationship, and the promotion of educational activities in the school environment were strategies used to address the problem. In this context, Latin American critical school psychology approaches developed the relational model of institutional analysis. A theoretical and practical approach that aims to understand and intervene in the institutional dynamics present in schools (Marinho-Araujo, 2014). This approach considers that the relationships established between the different actors in the institution (students, teachers, technical staff, managers, parents, etc.) are fundamental to the functioning and development of pedagogical practices. It is important to note that the relational model of institutional analysis is not limited only to school psychology, but is also used in other areas of psychology and the social sciences. It provides a broad and integrated view of institutions, considering individual and collective, cultural and structural aspects, in the search for a more just and equitable social transformation.

The theoretical framework is concerned with encompassing relational factors, avoiding the blaming of the student, the family, the school, or any of the poles involved in the pedagogical interaction. However, not all elements have the same weight in different school and cultural settings. For example, in the current economic and social scenario in Brazil, issues related to poverty, such as the need to work and contribute to the family income, still have a high weight, while pedagogical issues are secondary. It is expected that this scenario will change as policies to combat dropout advance, allowing us to focus on pedagogical, cultural, and psychological aspects.

While Early Warning Systems (EWS) have shown promising results in identifying students at risk of dropping out, there are some limitations and downsides to consider. One limitation of EWS is that they rely on data that may not capture the full range of factors contributing to a student’s dropout risk. For example, EWS may not account for mental health, social–emotional factors, or other non-academic challenges that can impact a student’s engagement and success in school (Mohanty and Dash, 2018). Another downside is that EWS can lead to the labeling and stigmatizing of students identified as at-risk. This labeling can create a self-fulfilling prophecy, as students identified as at-risk may believe that they cannot succeed in school (Davis and Dupper, 2004; Riley and Ungerleider, 2012).

In this sense, we are proposing to advance the conception of school-dropout warning systems from the articulation of multidimensional variables to a relational one (Davis and Dupper, 2004; Quin, 2017), where the system itself can be informed by how students and others school stakeholders are currently experiencing their schooling progression. While the before mentioned EWS construction presents a good case for developing systems that identify and predict school dropout, it is essential to recognize that they lack a more longitudinal and relational dimension to it (Murphy-Graham et al., 2021); for example, it is hard to understand from achievement data what is the relationship of the student with the school, its stakeholders, their community and how their parents think of the school system. With this in mind, there needs to be an instrument that can analyze and collect localized, personalized student information from both their peers and school personnel to ensure accurate prediction of at-risk students; the IAFREE using a relational model, might address precisely this issue by providing more subjective and complex data to integrate dropout early warning system.

## 3. Methods

### 3.1. IAFREE item development and item anchors

The IAFREE instrument was developed based on recommendations for scale construction best practices (American Educational Research Association, American Psychological Association, and National Council on Measurement in Education, 2014). The first step involved developing a working definition and structural framework of IAFREE grounded in an extensive literature review.

For example, in international EWS proposals, much is based on terminology such as the ABC’s typology (Attendance, Behavior incidents, and Course performance; UNICEF, 2018a,b), given that variables like frequent absenteeism for sickness, isolation from peers, etc., are often better predictors of dropout than intrinsic characteristics (Mac Iver and Mac Iver, 2009). Other guidelines considered were the Practioner’s Guide to implementing Early Warning Systems (Frazelle and Nagel, 2015), the Manual for School Prevention and Response Teams Towards Abandonment and Non-Registration in Compulsory Education (UNICEF, 2014), and the Policy and Practice Pointers for Enrolling all Children and Adolescents in School and Preventing Dropout (UNICEF, 2016). In the Brazilian context, we relied on the Public Policies to Reduce School Dropout by Young People (Instituto Ayrton Senna, 2017), which includes the best national practices and main valuable guidelines based on evidence for multiple factors contributing to early school leaving. In this sense, we are proposing a more accurate, personalized and faster assessment technique to overcome some of the current limitations of EWS, especially, because a psychometric model can point to better informed and fine grained data that generally governmental assessment cannot.

Specifically, based on this revision, five relational factors for predicting school dropout risk were created, as follows: I. Student-School, II. Student-School Professionals, III. Student-Family, IV. Student-Community, and V. Student–Student. The relational dimensions avoid the search for “culprits,” understanding that a complex phenomenon like this requires multidimensional measures and interventions, encompassing different education stakeholders in their interactions. Thus, each measure is related to the scope of the intervention. Suppose it is observed, for example, that the student-community relationship presents negative indicators. In that case, that indicates that the student does not perceive a clear connection between school education and his community, which constitutes an indicator of risk of evasion; the instrument already points to the need for interventions that promote these interactions. In a realistic view, the possible interventions encompass enabling the opening of the school to the community (e.g., using the court spaces, meetings, etc.), seeking partnerships for psychosocial care with institutional areas in the community, etc. These five relational factors are described below:

#### 3.1.1. I. Student-School (SSc)

This dimension consists of six items and encompasses the factors responsible for school dropout that refer to the relationships between the student and the material resources that constitute the school, such as teaching materials, furniture, and documents. This dimension indicates that the lack of structure and working conditions compromise the teaching and learning processes and, therefore, dropout rates (Ecker-Lyster and Niileksela, 2016; Churchill et al., 2021; Vinas-Forcade et al., 2021). This factor taps the following facets: student materials (SSc1: *“The kind of lunch served makes me thing about not going to school”*) and school materials (SSc2: *“I do not have an appropriate study space at home and that makes me think about not going to school”*).

#### 3.1.2. II. Student-school professionals

This factor consists of six items and encompasses the relationships between the student and the professionals who constitute the school’s daily life, such as managers, teachers, and other employees. Thus, the nature of the school climate (e.g., democratic relationships of collaboration or authoritarian relationships of competition) characterizes educational institution content organization and planning, evaluation, and decision-making (Uekawa et al., 2010; Marchbanks et al., 2014; Ecker-Lyster and Niileksela, 2016; King and Garcia, 2016; Nelson et al., 2016). This factor encompasses the following facets: pedagogical inflexibility (SP1*: “I thought about not going to school because of the amount of rules it has”*) and pedagogical quality (SP2: *“I thought about dropping out the school because the teachers miss class a lot”*).

#### 3.1.3. III. Student-Family (SF)

This dimension consists of six items and refers to how the student’s relationship with his family can influence the school dropout, given that the lack of family support is pointed as one of the factors that cause dropout (Ecker-Lyster and Niileksela, 2016; Rogers and Feller, 2016; Vinas-Forcade et al., 2021). Family support is understood here as the presence of the family and caregiver in school life, in events and meetings, concern for learning, understanding of the importance of school for the student’s future, help in performing school tasks, etc. (De Witte et al., 2013; Ecker-Lyster and Niileksela, 2016). This factor includes family support (SF1*: “Someone from my family, caregiver and/or guardian has already suggested that I leave school”*) and pregnancy, parenting, and household care activities (SF2: *“I’ve already missed school or failed to do homework because I had to help out at home cooking, cleaning, taking care of siblings”*).

#### 3.1.4. IV. Student-community (SC)

This factor consists of nine items and includes the social issues linked to learning difficulties and the dropout phenomenon. Social aspects are understood here as a socioeconomic vulnerability that produces the need for work in childhood and adolescence, the lack of opportunity to study at an appropriate age, the presence of crime and access to substances in the community, as well as the presence of themes and community culture in school and the construction of networks with other sectors of the neighborhood (Dever and Raines, 2013; Rogers and Feller, 2016; Churchill et al., 2021; Vinas-Forcade et al., 2021). The facets of this factor are socio-educational measures and context of violence (SC1: *“There is suspicion and/or indications that this student may have been or is being the victim of violence”; item registered by a school professional*), accessibility/school attendance (SC2: *“I’ve thought about leaving school because I do not study the history or characteristics of my community/city there”*), and school-community distancing (SC3: *“This student is missing more than is acceptable”*; *item registered by a school professional*).

#### 3.1.5. V. Student–Student (SSt)

This dimension consists of nine items and taps concerns about the learning process’s affective, psychological, and cognitive aspects and dropout. In addition, this factor assesses the characterization and quality of affective bonds between students at school, as well as the presence of violence, disruptive behaviors, illnesses, and mental disorders as factors linked to dropout (Snyder and Cooper, 2015; Churchill et al., 2021), as well as the amount of stress, anxiety, negative feelings and emotions associated with the experiences of performing school tasks and activities (Dever and Raines, 2013; Nelson et al., 2016; Vinas-Forcade et al., 2021). The facets of this factor are the meaning of graduation/student engagement (SSt1: *“I’ve thought about leaving school because it does not deal with current affairs”*), emotional and affective aspects/socioemotional health (SSt2: *“I’ve thought about leaving school because my schoolmates do not treat me well”*), and failures and age-grade distortion (SSt3: *“This student has a grade/age gap, not being in the expected grade for his age group”*; *item registered by a school professional*).

The initial item pool for IAFREE consisted of 50 items based on theoretical issues and previous empirical evidence. We followed Kline’s (2015) guidelines for developing scale items. In sum, the construction of the items was based on several criteria, including (a) clarity of the item, (b) ease of reading the item, (c) other ways the item might be inquired, and (d) what the item is measuring. Further, to establish content evidence for IAFREE (i.e., theoretical analysis), qualified judges were enrolled to analyze the items considering the following indexes: (a) content relevance and (b) content representations (i.e., if the items measure the relevant domains of the construct (Church and Waclawski, 2007). In sum, the expert review included six scholar-researchers. The selection criteria included their research fields in educational themes and psychometrics (adaptation and development of measures). After the feedback from experts and the revision of ambiguous items, the experimental version of IAFREE consisted of a 36-item scale (six items for the first, second, and third factors; nine items for the fourth and fifth factors).

The third step consisted of a pilot study that implies a prior instrument administration in a small sample reproducing the sample/target-population characteristics (Gudmundsson, 2009). The sample/target population was composed of 58 respondents. Following the Standards for Educational and Psychological Testing (American Educational Research Association, American Psychological Association, and National Council on Measurement in Education, 2014), we aimed to identify some test features, including instructions, time limits, item response formats, and item response options. Specifically, this step was necessary as the instrument is answered by both the student and school professionals (e.g., teacher and school manager). The records of the answers were made digitally through a computer system. For example, in dimension V. Student–Student, the student answers the item “I’ve thought about dropping out of school because it does not prepare me for the jobs I want in the future.” (facet: Meanings of Schooling/Engagement). The school manager answers the item, “This student has a grade/age gap, not waiting in the grade expected for his age group.” (facet: Failures and age-grade interruption). As a result of this stage, it was decided to offer specific training for school administrators to adequately complete the online questionnaire, including school and sociodemographic variables.

### 3.2. Participants

A sample of 15,924 Brazilian high school and middle school students participated in this study (Table 1). Regarding sociodemographic variables, the following characterizations were observed: Gender (boys = 8.260; girls = 7.664), Ethnicity (Black = 1,639; Brown = 11.602; White = 2.233; Indigenous people = 282; Asian = 168), Geographic location (urban area = 7.954; rural area = 7.644, Indigenous community = 211; Quilombolas Communities = 116). About the other variables, most of them were: school failure (Never, *n* = 12.785), marital status (single, *n* = 15.709), Mother/caregiver’s education (high school degree, *n* = 3.656), Father/caregiver’s education (middle school degree, *n* = 2.916). Finally, most of the sample is distributed among the middle school levels (*n* = 15.825; high school level, *n* = 99). This was a non-probabilistic sample, with individuals voluntarily deciding to participate (i.e., snowball sampling; Dusek et al., 2015). All the terminology was inspired by the nationwide survey used by the government institute responsible for Brazil’s population census, the IBGE.

**Table 1:** Sociodemographic characteristics of the sample (*n* = 15.924).

Characteristics		*F*	%
*Sex*			
	Male	8.260	51.9%
	Female	7.664	48.1%
*Suspicion/Confirmation that the student works*			
	Yes	1.676	10.5%
	No	14.248	89.5%
*Residence location*			
	Rural zone	7.644	48.0%
	Urban zone	7.953	49.9%
	Indigenous community	211	1.3%
	Quilombola community	116	0.7%
*School failure*			
	Never	12.785	80.3%
	Once	2.039	12.8%
	Twice	766	4.8%
	Three times or more	334	2.1%
*Marital status*			
	Single	15.709	98.6%
	Married	125	0.8%
	Divorced	7	0.01%
	Widow(er)	5	0.01%
	Stable union	78	0.5%
*Race/ethnicity*			
	Black	1.639	10.3%
	Brown	11.602	72.9%
	White	2.233	14.0%
	Indigenous	282	1.8%
	Asian	168	1.1%
*Monthly family income*			
	None	2.018	12.7%
	Up to minimum wage (up to R$ 1.212)	8.082	50.8%
	From 1 to 3 minimum wages (from R$ 1.212 to R$ 3.636)	4.843	30.4%
	From 3 to 6 minimum wages (from R$ 3.636 to R$ 7.272)	781	4.9%
	From 6 to 9 minimum wages (from R$ 7.272 to R$ 10.908)	135	0.8%
	More than 9 minimum wages (more than R$ 10.908)	65	0.4%
*Mother/caregiver’s education*			
	From 1st to 4th grade of Elementary School	2.147	13.5%
	From 5th to 8th grade of Elementary School	3.538	22.2%
	High School	3.656	23.0%
	University education	1.007	6.3%
	Specialization	218	1.4%
	Didn’t study	325	2.0%
	Uninformed	5.033	31.6%
*Father/caregiver’s education*			
	From 1st to 4th grade of Elementary School	2.249	14.1%
	From 5th to 8th grade of Elementary School	2.916	18.3%
	High School	2.410	15.1%
	University education	490	3.1%
	Specialization	123	0.8%
	Didn’t study	518	3.3%
	Uninformed	7.218	45.3%
*School grade*			
	Fifth Year of Elementary School	73	0.5%
	Sixth Year of Elementary School	4.629	29.1%
	Seventh Year of Elementary School	4.072	25.6%
	Eighth Year of Elementary School	3.794	23.8%
	Ninth Ano do Ensino Fundamental	3.257	20.5%
	First Year of High School	58	0.4%
	Second Year of High School	16	0.1%
	Third Year of High School	25	0.2%

### 3.3. Measure

Relational Factors for the Risk of School Dropout Scale (IAFREE). This is a 36-item measure that assesses the relational factors for the risk of school dropout (See Table 2 for a description of the items) in both middle and high school populations. The IAFREE consisted of five subscales that comprise the following elements: Student-School (two facets: SSc1/SSc2), Student-Professionals (two facets: SP1/SP2), Student-Family (two facets: SF1/SF2), Student-Community (three facets: SC1/SC2/SC3), and Student–Student (three facets: SSt1/SSt2/SSt3). Thus, for the IAFREE, we expect a higher-order internal structure with 12 first-order risk facets and 5 s-order factors. All items were rated by respondents using seven response categories ranging from 1 (completely disagree) to 7 (completely agree).

**Table 2:** Description of IAFREE items.

Items	Description
S1	Sinto tristeza ou estou deprimido (a) e isso me faz pensar em abandonar a escola.
S2	Sinto que sou incapaz de concluir meus estudos e por isso penso em abandonar a escola.
S3	O tipo de merenda servido me faz pensar em não ir pra escola.
S4	Pensei em me afastar da escola por não ter equipamentos adequados para lidar com as condições do clima (calor, frio e chuvas).
S5	Já pensei em abandonar a escola, pois ela não me oferece possibilidades de melhoria das minhas atuais condições de vida.
S6	Já pensei em deixar a escola pois ela não trata dos assuntos atuais
S7	Já pensei em abandonar a escola por ter tido e/ou ter um problema de saúde.
S8	Já pensei em deixar a escola pois nela não estado a história ou características da minha comunidade/cidade.
S9	Não tenho um espaço apropriado de estudos em casa e isso me faz pensar em me afastar da escola.
S10	Já pensei em abandonar a escola pois ela não me prepara para os empregos que desejo no futuro.
S11	Já pensei em deixar a escola porque ela não respeita a religião que eu pratico.
S12	Pensei em abandonar a escola pois os (as) professores (as) faltam muito.
S13	Pensei em me afastar da escola pela quantidade de regras que ela tem.
S14	Já pensei em deixar a escola porque ela é frequentemente alvo de violência (vandalismo, assaltos, pichações, toque de recolher e etc).
S15	Já faltei à escola ou deixei de fazer lição de casa por ter que ajudar em atividades de casa (cozinhar, limpar, cuidar de irmãos).
S16	Alguém da minha família, cuidador (a) e/ou responsável já sugeriu que eu deixasse a escola.
S17	Pensei em abandonar a escola por não ter dinheiro para ir ou pela dificuldade que encontro no percurso/caminho para chegar até a escola.
S18	Não poder realizar atividades artísticas ou culturais na escola é algo que me faz querer me afastar.
S19	Pensei em abandonar a escola porque as salas têm mais estudantes do que os professores conseguem dar atenção.
S20	Pensei em abandonar a escola pois as aulas são repetitivas e cansativas.
S21	Já pensei em abandonar a escola por ter tido e/ou ter um problema de saúde na família.
S22	Pensei em abandonar a escola por não poder praticar os esportes que eu queria.
S23	Não poder fazer uso da internet na escola é algo que me faz querer me afastar.
S24	Já pensei em deixar a escola porque meus/minhas colegas de escola não me tratam bem.
PA1	Este estudante está faltando mais do que o aceitável.
PA2	Este estudante não tem fardamento (uniforme escolar) e/ou sapatos e vestimenta adequada.
PA3	Existe suspeita e/ou indícios de que este estudante pode ter sido ou estar sendo vítima de violência.
PA4	Existe suspeita e/ou comprovação de que este estudante possui dificuldades de aprendizagem.
PA5	Existe suspeita e/ou indícios de que este estudante cumpriu e/ou está cumprindo alguma medida socioeducativa.
PA6	Este estudante não apresenta rendimento esperado, tirando notas abaixo da média nas disciplinas de Português e/ou matemática.
PA7	A mãe ou cuidador de referência deste estudante não concluiu o ensino básico.
PA8	Este estudante não tem material escolar (lápis, caderno, etc.).
PA9	Este estudante apresenta defasagem série/idade, não estando na série esperada para sua faixa etária.
PA10	Existe suspeita e/ou indícios de que este estudante pode estar envolvido(a) com uso e/ou com o tráfico de substâncias.
PA11	Os familiares e/ou cuidadores deste estudante não comparecem à escola quando solicitados.
PA12	Existe suspeita e/ou indícios de que esta estudante, ou alguma companheira (no caso dos estudantes do sexo masculino) engravidou durante o ano letivo e tenha abandonado a escola.

### 3.4. Procedure

The middle and high schools were selected by the convenience criterion. The educational institutions were recruited through e-mail provided by the local government’s websites, and the individuals answered a web-based questionnaire. The aims and procedures were described, and the survey link for their participation was provided. In the first step (self-report measure), students completed surveys during school hours for approximately 10 min using a handheld digital device. In the second stage (third-person measure), the school manager answered the items referring to the student. Yet, consent from each student was obtained at the beginning of each online survey. All participants were informed that the study was voluntary. The Ethics Committee has approved the research project from a public university in Brazil (Process N° n. 5.407.594, Healthy Sciences Centre).

### 3.5. Data analysis

Statistical analyses were performed using R software (R Development Core Team, 2021). Through the Lavaan package (Rosseel, 2012), We performed Confirmatory Factor Analysis (CFA) to assess the adequacy of the proposed model of the IAFREE, based on Weighted Least Squares Mean and Variance-Adjusted (WLSMV; Muthén and Muthén, 2014), implemented by polychoric correlations, because WLSMV is designed explicitly for ordinal data (Li, 2016). For the CFA interpretation, the following fit indicators were used (benchmarks of good fit; Tabachnick and Fidell, 2013; Kline, 2015): Chi-square to the degrees-of-freedom ratio (S-B*χ*^2^/*df*), which should be lower than 3; Comparative Fit Index (CFI) and Tucker-Lewis index (TLI), which should be greater than 0.90; and Standardized Root Mean Square residual (SRMR) and root mean square error of approximation (RMSEA), which should present values between 0.05 and 0.08. Finally, reliability was calculated by the Raykov’s Composite Reliability (CR) statistic (Raykov, 1997), and through Cronbach’s alpha, and McDonald’s omega (*ω*), with recommended values over 0.70 (Kline, 2013).

In the second step, Item Response Theory analysis was performed. Again, R was used to calculate the individual parameters of the items through mirt package (Chalmers, 2012) to assess each item’s threshold, discrimination, and informative curve. Due to the polytomous nature of the instrument, the Graded Response Model (Samejima, 1969) was implemented.

## 4. Results

**Figure 1:**
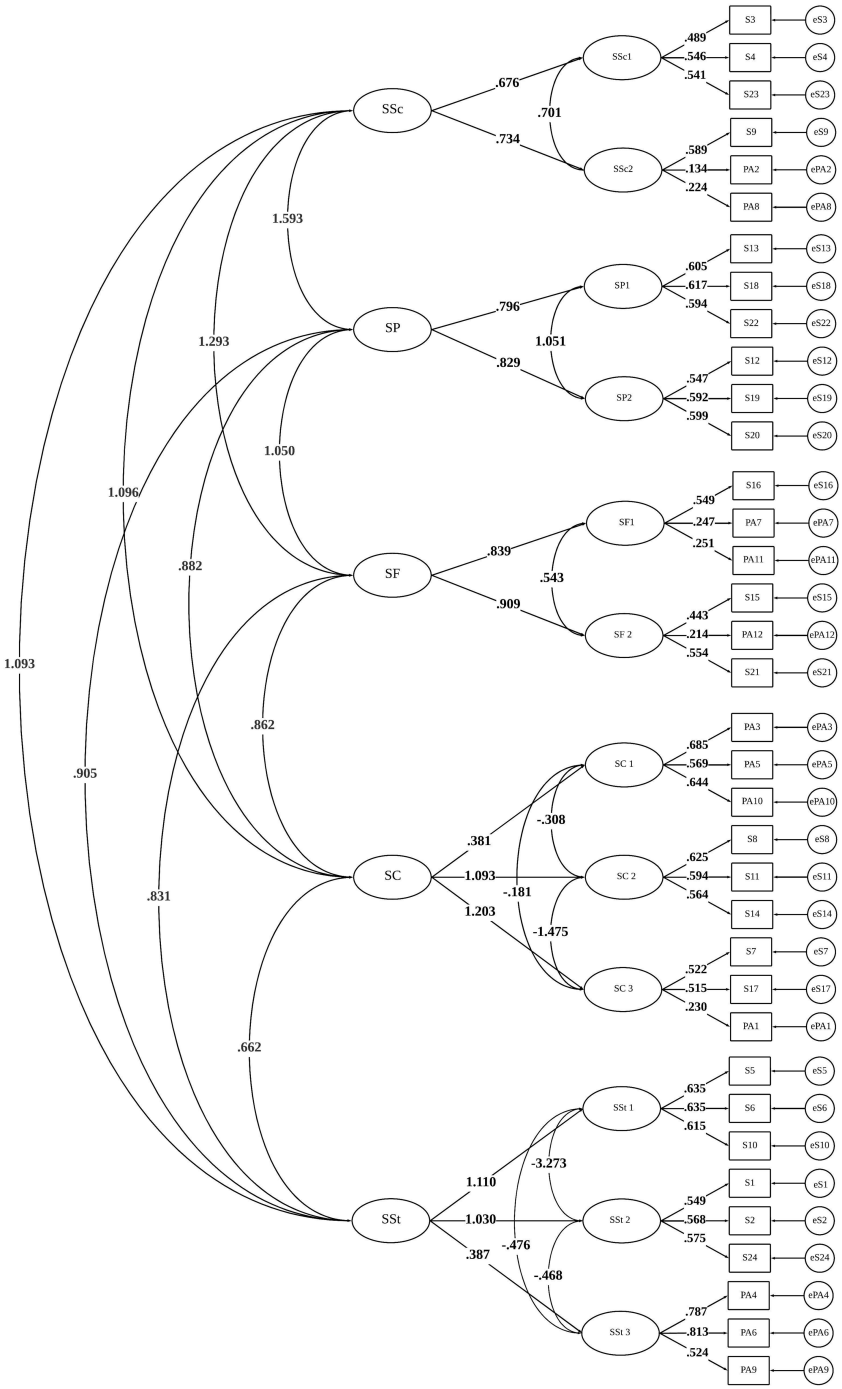
Factorial structure of the IAFREE-36.

Firstly, a CFA was performed to check the model fit of the higher-order factor structure. Results indicated acceptable fit: χ^2^ (568) = 17,576,592, *p* < 0,001, CFI = 0,905, TLI = 0,894, RMSEA = 0,043 (IC95% = 0,043–0,044), and SRMR = 0,064. All the factorial loadings (lambdas) were different from zero (λ ≠ 0; z > 1.96, *p* < 0.05), ranging from 0.13 (item SP2) to 0.68 (item SP3; See Table 3; Figure 1). Concerning reliability coefficients (See Supplementary file), acceptable results were observed in general. However, among the 12 risk factors, the SSc2 (Student-School dimension), SF1 and SF2 (Student-Family dimension), and SC3 (Student-Community dimension) presented indicators below the expected (< 0.70). It is worth noting that these dimensions include items answered by both the student and school professionals. When we handled the reliability indices separately for student responses and those registered by school professionals, the former presented excellent internal consistency (*α* = 0.89, *ω* = 0.88, C.R = 0.90), and the latter presented indicators above 0.70 (*α* = 0.76, *ω* = 0.76, C.R = 0.78).

**Table 3:** Factorial loadings and the parameters of the items of IAFREE.

Dim	Factor	Items (English)	*a*	*b_x_*	*λ*
SSc		The kind of lunch served makes me think about not going to school (S3)	2.264	1.545	0.489
	SSc1	I thought about leaving school because I did not have adequate equipment to deal with weather conditions (S4)	1.784	1.930	0.546
		Not being able to use the internet at school makes me want to stop going (S23)	1.557	1.562	0.541
		I do not have an appropriate study space at home and that makes me think about not going to school (S9)	0.815	3.758	0.589
	SSc2	This student does not have a uniform (school uniform) and/or appropriate shoes and clothing (PA2)	1.640	2.109	0.134
		This student does not have school supplies (pencil, notebook, etc.) (PA8)	6.960	1.922	0.224
SP		I thought about not going to school because of the amount of rules it has (S13)	1.992	1.624	0.676
	SP1	Not being able to do artistic or cultural activities at school makes me want to stop going to school (S18)	2.776	1.809	0.734
		I thought about dropping out the school because I could not play the sports I wanted (S22)	2.565	1.565	0.701
		I thought about dropping out the school because the teachers miss class a lot (S12)	2.355	2.010	0.605
	SP2	I thought about dropping out the school because the classrooms have more students than teachers can handle (S19)	2.507	1.705	0.617
		I thought about leaving the school because the classes are repetitive and tiring (S20)	1.900	1.549	0.594
SF		Someone from my family, caregiver and/or guardian has already suggested that I leave school (S16)	0.867	3.731	0.547
	SF1	This student’s mother or primary caregiver did not complete basic education (PA7)	0.944	1.123	0.592
		This student’s family members and/or caregivers do not attend school when requested (PA11)	2.283	1.726	0.599
		I’ve already missed school or failed to do homework because I had to help out at home (cooking, cleaning, taking care of siblings) (S15)	1.093	1.272	0.796
	SF2	There is suspicion and/or evidence that this student or a partner (in the case of male students) became pregnant during the school year and dropped out of school (PA12)	0.895	5.301	0.829
		I’ve thought about dropping out of school because I had and/or have a health problem in the family (S21)	3.331	1.554	1.051
SC		There is suspicion and/or indications that this student may have been or is being the victim of violence (PA3)	4.412	0.923	0.549
	SC1	There is suspicion and/or evidence that this student has fulfilled and/or is complying with some socio-educational measure (PA5)	4.036	0.854	0.247
		There is suspicion and/or indications that this student may be involved with substance use and/or trafficking (PA10)	1.118	2.518	0.251
		I’ve thought about leaving school because I do not study the history or characteristics of my community/city there (S8)	2.431	2.013	0.443
	SC2	I’ve thought about leaving school because it does not respect the religion I practice (S11)	3.074	1.950	0.214
		I’ve thought about leaving the school because it is often the target of violence (vandalism, robberies, graffiti, curfew, etc.) (S14)	2.062	2.032	0.554
		I’ve thought about dropping out of school because I had and/or have a health problem (S7)	1.786	1.976	0.839
	SC3	I thought about dropping out of school because I did not have the money to go or because of the difficulty I find on the way to get to school (S17)	2.569	1.986	0.909
		This student is missing more than is acceptable (PA1)	0.690	3.646	0.543
SSt		I’ve thought about dropping out of school, because it does not offer me possibilities to improve my current living conditions (S5)	2.488	1.814	0.685
	SSt1	I’ve thought about leaving school because it does not deal with current affairs (S6)	2.939	1.735	0.569
		I’ve thought about dropping out of school because it does not prepare me for the jobs I want in the future (S10)	2.244	1.640	0.644
		I feel sad or depressed and it makes me think about dropping out of school (S1)	2.479	1.496	0.625
	SSt2	I feel that I am unable to complete my studies and so I think about dropping out of school (S2)	2.450	1.573	0.594
		I’ve thought about leaving school because my schoolmates do not treat me well (S24)	1.397	1.858	0.564
		There is suspicion and/or proof that this student has learning difficulties (PA4)	4.209	0.927	0.522
	SSt3	This student does not show the expected performance, obtaining grades below average in the Portuguese and/or Mathematics subjects (PA6)	4.199	0.848	0.515
		This student has a grade/age gap, not being in the expected grade for his age group (PA9)	1.414	1.821	0.230

*a* = discrimination parameter; *b_x_* = difficulty average value; *λ* = standardized loadings; S = Student answer; PA = Answer registered by school professional.

Next, we performed an IRT to evaluate the IAFREE-36 scale further. Then, we analyzed the capacity of the items to discriminate between people and spread in the thresholds and information (see Supplementary file). Firstly, in the Student-School dimension, for the SSc1 factor, the capacity of items to discriminate was strong (Mean = 1.87, SD = 0.36). The most discriminative item was S3 (*a* = 2.26). For the SSc2, the capacity of items to discriminate was very strong (Mean = 3.14, SD = 3.33). The most discriminative item was PA2 (*a* = 1.64). In the second dimension, Student-School Professionals, for the SP1 factor, the capacity of items to discriminate was very strong (Mean = 2.44, SD = 0.40). The most discriminative item was S18 (*a* = 2.77). Finally, for the SP2 factor, the general discrimination indices were very strong (Mean = 2.25, SD = 0.31), and S19 was the item with the highest discrimination indices (*a* = 2.50).

In the third dimension, Student-Family, for the SF1 factor, the capacity of items to discriminate was strong (Mean = 1.36, SD = 0.79). The most discriminative item was S11 (*a* = 2.83). For the SF2 factor, the general discrimination indices were very strong (Mean = 1.77, SD = 1.35), and S21 was the item with the highest discrimination indices (*a* = 3.31).

The fourth dimension, Student-Community, comprises the following risk factors: SC1 (very high discrimination; Mean = 3.19, SD = 1.80), SC2 (very high discrimination; Mean = 2.52, SD = 0.51), and SC3 (high discrimination; Mean = 1.68, SD = 0.94). The items with the highest discrimination indices were PA3 (a = 4.41), S11 (*a* = 3.07), and S17 (*a* = 2.56), respectively.

Finally, for the fifth dimension, Student–Student, the following discrimination was observed: SSt1 (very high discrimination; Mean = 2.56, SD = 0.35), SSt2 (very high discrimination; Mean = 2.11, SD = 0.61), and SSt3 (very high discrimination; Mean = 3.27, SD = 1.61). The items with the highest discrimination indices were S6 (*a* = 2,93), S1 (*a* = 2,47), and PA4 (*a* = 4,20), respectively.

With regard to the difficulty parameter (see Table 3 and Supplementary file), for the Student-School dimension, both SSc1 and SSc2 presented very difficult items (*b* < 1.28). The same results were observed for the two Student-School Professionals’ dimension risk factors. For the third dimension, Student-Family, the items from two factors presented items varying from difficult to very difficult items. For the Student-Community dimension, the results showed items varying from moderate to very difficult items (SC1) and very difficult items (SC2). Finally, for the fifth dimension, the risk factors of SSt1 and SSt2 presented items with very difficult items. For the SSt3 risk factor, the difficulty level of items varies from difficult to very difficult.

**Figure 2:**
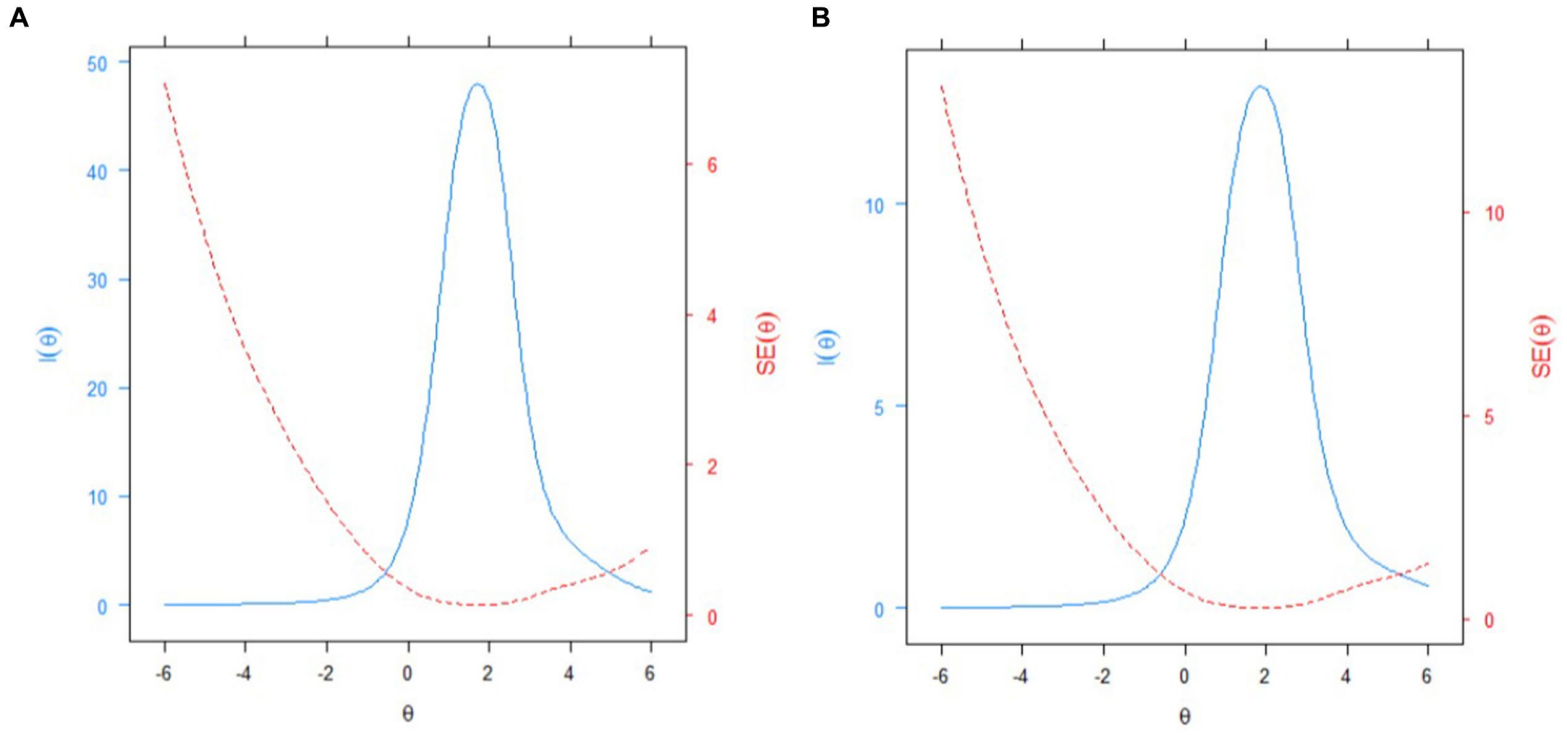
Test information curves: **(A)** IAFREE-36, and **(B)** IAFREE-12.

In the next step, we selected from IRT’s analysis the 12 items with psychometric quality information to achieve a brief-report measure (IAFREE-12: S3, PA2, S18, S19, PA11, S21, PA3, S11, S17, S6, S1, and PA4). Specifically, these items were derived from the indices of discrimination (Table 4). For CFA analysis, the goodness-of-fit indices obtained a reasonable fit for the unidimensional model *χ*^2^ (54.000) = 1651.869, *p* < 0.001, CFI = 0.902, TLI = 0.880, RMSEA = 0.043 (95%CI = 0.041–0.045), and SRMR = 0.053. Furthermore, the reliability indices were considered acceptable (*ω* = 0.70, *α* = 0.70, C.R. = 0.71). Finally, with regard to the test information curves, the reduction does not compromise the psychometric information, covering approximately the same latent trace portion (Figure 2).

**Table 4:** Factorial loadings and the parameters of the items of IAFREE-12.

Dimensions	Factors	Items (English)	*a*	*b_x_*	*λ*
SSc	SSc1	The kind of lunch served makes me think about not going to school (S3)	1.44	1.93	0.41
	SSc2	This student does not have a uniform (school uniform) and/or appropriate shoes and clothing (PA2)	0.80	3.41	0.12
SP	SP1	Not being able to do artistic or cultural activities at school makes me want to stop going to school (S18)	2.61	1.84	0.57
	SP2	I thought about dropping out the school because the classrooms have more students than teachers can handle (S19)	2.20	1.79	0.56
SF	SF1	This student’s family members and/or caregivers do not attend school when requested (PA11)	0.90	2.99	0.19
	SF2	I’ve thought about dropping out of school because I had and/or have a health problem in the family (S21)	2.00	1.85	0.50
SC	SC1	There is suspicion and/or indications that this student may have been or is being the victim of violence (PA3)	1.14	3.77	0.22
	SC2	I’ve thought about leaving school because it does not respect the religion I practice (S11)	2.54	2.03	0.53
	SC3	I thought about dropping out of school because I did not have the money to go or because of the difficulty I find on the way to get to school (S17)	2.50	1.98	0.51
SSt					
	SSt1	I’ve thought about leaving school because it does not deal with current affairs (S6)	2.28	1.88	0.56
	SSt2	I feel sad or depressed and it makes me think about dropping out of school (S1)	1.39	1.96	0.44
	SSt3	There is suspicion and/or proof that this student has learning difficulties (PA4)	0.65	2.38	0.22

*a* = discrimination parameter; *b_x_* = difficulty average value; *λ* = standardized loadings; S = Student answer; PA = Answer registered by school professional.

## 5. Discussion

School dropout or early school leaving is viewed as an inefficiency of educational systems and a significant and multifaceted problem in most countries (Gonzalez-Rodriguez et al., 2019). Recent methodologies have focused on both student- and school-level variables in order to provide a more in-depth assessment of the underlying mechanisms associated with this phenomenon (Wood et al., 2017). As understanding the risk factors provides a starting point to tackle school dropout (Lansford et al., 2016), here we introduced an instrument to measure a five-factor model of risk for school dropout derived from best practices and empirical national and international evidence: the IAFREE. The current initial psychometric evaluation of this measure appears to be promising.

The IAFREE took a novel approach to risk factors for early school leaving. In sum, this perspective unifies in a single instrument different dimensions of SD and the interrelation between them. Thus, from a relational and ecological perspective, this instrument can predict the complex relational system that leads to SD. This instrument seeks to overcome flaws identified in the current area, especially the dissociation between analytical and systemic approaches (Wood et al., 2017; Kearney, 2021). Defining systemic approaches as those that cover broad contexts and perspectives (e.g., social context and educational policies in a relational way: school-family and family-community relationship). The latter model focuses on individual issues, such as student characteristics, and more immediate components, such as absenteeism (Kearney, 2021). Integrating approaches can help fill gaps in understanding, connecting research and decision-making, theory and public policy, prediction, and intervention. Longitudinal monitoring of the evolution of these dimensions will make it possible to understand how the students’ trajectory follows directions that lead to dropout or the development of protective factors (Wood et al., 2017).

Regarding psychometric properties, across several technical procedures, we provided empirical evidence for the IAFREE in the Brazilian context (i.e., both a full and a brief measure). In the first step, encompassing theoretical issues, qualified judges analyzed the relationship between the content of the items and the construct they were intended to measure (American Educational Research Association, American Psychological Association, and National Council on Measurement in Education, 2014). Thus, we intended to ensure that the content domains were associated with the inferences from test items. Following guideline steps, we executed a pilot study addressing the suitability of items (e.g., their meaning, difficulty, etc.). Specifically, considering that the IAFREE includes some items answered by school professionals, we evaluated the test instructions, given that the administration and scoring may also be important to content-based evidence (American Educational Research Association, American Psychological Association, and National Council on Measurement in Education, 2014).

At the empirical stage, the confirmatory factor analysis showed that the higher-order model for the full measure (IAFREE-36) presented adequate fit indices (Hair et al., 2014). These results are in line with previous research indicating the phenomenon of SD is better captured by multiple domains (e.g., individual, family, school, and community factors) and their respective subdomains (e.g., school: school structure, school resources, etc.; Hammond et al., 2007). With regard to reliability coefficients for the IAFREE-36, the overall dimensions presented acceptable to good internal consistency (George and Mallery, 2003); the dimensions of Student-School and Student-Family did not reach the recommended cut-off points (Kline, 2013). Thus, future studies should assess this indicator through an alternative method: test–retest reliability (i.e., associating pairs of scores from the same student on two different administrations of the same instrument; Cohen and Swerdlik, 2018). As the risk factors for SD are likely to vary, it may be rational to accept the variance over time (American Educational Research Association, American Psychological Association, and National Council on Measurement in Education, 2014) and estimate it based on error variance sources such as questionnaire sampling, students, occasions, raters, etc.

Through the paradigm of the IRT, discrimination, and difficulty parameters were generated. In sum, most of the items across all the five dimensions presented high to very discrimination levels that is an adequate ability to differentiate people according to each correspondent risk factor for SD (Baker, 2001), ensuring that the test may be able to map subjects along the continuum of the latent trait (Embretson and Reise, 2000). Also, we found that the overall items are not easily endorsed, meaning that they presented high levels for the difficulty parameter (Pasquali, 2020). In other words, the item threshold analysis (i.e., difficulty parameter) indicates that the participants needed high levels of the latent trait to endorse the risk factors for SD. This pattern of results is common for clinical and personality variables, and it is classified as a “quasi-trait” (Reise and Waller, 2009), displaying a unipolar construct (i.e., appropriate in only one direction) that means scores variation at the low end of the continuum is less informative. Thus, the low continuum of the risk factors for SD does not measure, for example, protection factors for SD, but rather the relative lack of risk factors components (Breivik and Olweus, 2015).

Further, based on the IRT results, we selected the 12 most informative items to comprise the IAFREE (IAFREE-12) short version. The CFA supported the unidimensional structure of the IAFREE-12 (Kline, 2015). In addition, these items maintained adequate indicators of discrimination and reliability without losing psychometric information (Baker, 2001) compared to the full-scale version. In sum, a brief scale consists of a self-report measure that permits the more effective measurement of a psychological variable compared with another scale with comparable psychometric evidence but more items (Kemper et al., 2019). The use of short instruments for psychological assessment has increased (Kruyen et al., 2013). The main advantages of short measures are associated with reducing costs (e.g., in epidemiological surveys), improving the participation of subjects (Edwards et al., 2004), and avoiding fatigue, which might result in higher data quality (Credé et al., 2012).

In light of these results, the benefits of having a multidimensional measure for evaluating relational factors for predicting school dropout (SD) are associated with multiple possible target actions. Firstly, this tool enables a student early alert system that can be viewed as a proactive measure to enhance the provision of support and assistance (Atif et al., 2020). Second, it also allows for allocating resources effectively within educational systems (Limone and Toto, 2022). The third point is related to an essential element: evaluating policies and programs. The continuous evaluation allows insights to support public policy decision-making and to develop different strategies to reach out to it, such as raising community awareness and educating the public in an attempt to gain support for this topic (Babinski et al., 2016) and developing predictive models for dropout in school systems through computerized tools, etc. (Rodríguez et al., 2023). In addition, as there is increasing interest regarding the ethical and political aspects of education, a fourth benefit of an instrument that taps relational factors for predicting SD is associated with promoting equity in education (Appels et al., 2022). Fifth, this measure, within a school dropout early warning system, can improve long-term education since school dropout has multiple significant costs to society (Reinke et al., 2022). Finally, the brief-report measure (IAFREE-12) is a tool for screening and assisting school managers in placing timely decision-making and interventions (Reinke et al., 2022). This is an accessible measure and can be valuable in large research projects in order to assess multiple antecedents and consequences of risk factors for SD (Edwards et al., 2004).

Compared to the IAFREE type of data collected and analyzed, other models may fall short with potential downsides. Despite the ABC or UNESCO model’s comprehensive framework for assessing school dropout, it fails to consider the subjective interaction between students and teachers. Data gathered from this model strictly looks at general factors, especially econometric ones, rather than the factors that can emotionally impact those who leave school—such as family difficulties or community pressures, which can be difficult to quantify. The ABC model, while being convenient and simple to use, has its limits. The system of early warnings relies heavily on objective data points, like attendance, behavior, and course performance, unable to truly account for emotional distress or disconnection with school and others. On the other hand, the relational model we adopt overcomes these restraints by analyzing subjective relationship metrics between students and teachers, in addition to the school, their peers, family, and the community. This additional data layer has enabled a more intimate analysis of why students might be at risk of dropping out, allowing schools to tailor support to those who a more objective measure might overlook. Furthermore, the IAFREE model considers the views of both parties (schoolteacher or dean and students), providing a holistic view of student experiences within the school.

## 6. Conclusion

At-risk students must have an efficient system that renders their predicaments more visible, allowing stakeholders to spot and subsequently offers targeted bolstering to these learners. Without this system, students may go unseen and unsupported, increasing their chances of discontinuing school. By capturing the personal experiences and assessments of pupils and teachers, Early Warning Systems informed by instruments such as the IAFREE relational model can detect learners who may not be deduced through unbiased means only, thus supplying a more accurate portrayal of the elements resulting in school dropout.

Stakeholders, such as instructors, counselors, and superiors, can act while availing of the knowledge given by the early warning system to render concentrated interventions and assistance to vulnerable learners. If, for instance, the system flags a student battling with a feeling of detachment from school and comrades, stakeholders can interact to provide guidance or counseling to aid the student perceive more connected and involved. Should the system point out a student suffering from familial or communal hindrances, stakeholders can intercede to join the student or their family to the befitting resources and support amenities.

Our model has a key advantage: it informs stakeholders specifically and in a timely manner about the dimensions and risk factors that require action. Thanks to the IAFREE-informed EWS, stakeholders, especially politicians and school principals, can closely examine school-level and student-level risk factors that pose a greater risk. For instance, based on the IAFREE assessment, school principals can learn that the most prominent dimension for dropout risk in their school is related to student–student relationships, specifically socioemotional issues like bullying. Additionally, they can be informed about a specific student who is experiencing a disconnection between their culture and the school system, which affects their prospects for the future, particularly for immigrant students. This level of detailed information is rarely available in EWS, but the model presented in this paper overcomes this well-known limitation. Overall, an early warning system like IAFREE model allows stakeholders to comprehend the elements contributing to school desertion more precisely and complexly. By recognizing and offering targeted aid to susceptible students, stakeholders can take the necessary steps to counter dropout and motivate academic consistency for every pupil.

### 6.1. Limitation and future direction

Despite the observed results and the psychometric quality of the IAFREE, we believe that there are some limitations associated with this study. Firstly, the samples used are non-probabilistic; therefore, generalizability may be limited. Thus, future agendas should consider replication analyses of the IAFREE with a more diverse sample of school-going children and adolescents due to the disparities between five Brazilian regions and institutions regarding access, quality, and education funding (Zapata et al., 2015). In addition, beyond the Brazilian context, future studies should test the IAFREE structural model in other countries for cross-cultural purposes (e.g., cross-cultural evaluation of educational systems, large-scale international comparative studies of educational indicators, etc.; International Test Commission, 2017).

It is worth noting that all the statistical analysis performed relied on Classical Test Theory (i.e., EFA, CFA). With respect to Item Response Theory (van der Linden, 2016), we only evaluated the item’s difficulty and discrimination properties. In addition, due to the previous evidence that some variables (e.g., gender, race, physical abuse, and affiliating with antisocial peers during adolescence; Lansford et al., 2016; Piscitello et al., 2022) affect dropout rates, future research may look into differential item functioning, through IRT’s models, to eliminate potential sociodemographic biases in responses (Janssen, 2018).

Yet, based on psychometric issues, relative representative samples will allow future investigations to establish reference norms concerning age groups (i.e., norm-referenced; Cohen and Swerdlik, 2018). This future objective will also provide a reliable and valid tool for screening school dropout risk in middle and high school students (i.e., through ROC curve), leading to an evidence-based decision-making process (Gulliford, 2015). Additionally, considering the growing interdependence between educational practices and internet-based tools (Wang, 2022), future avenues may address the construction of a national digital platform, including IAFREE. These efforts contribute to novel initiatives in education, such as artificial intelligence and machine learning, but in line with a critical perspective by ensuring that the use of novel technologies in testing means accuracy, accessibility, engagement, and fair assessments (International Test Commission and Association of Test Publishers, 2022).

Furthermore, we did not access the evidence based on relationships to other variables. For instance, new research should establish the nomological network of the IAFREE. In terms of antecedents, it is also important to test variables such as absenteeism, failure to be promoted to the next grade level, being detached in the classroom (Neild and Balfanz, 2006), ongoing failure, retention, school policies, and being of age to leave the system finally (Bradshaw et al., 2008; Bowers, 2010), academic achievement, retention, sex, family socioeconomic status, and extracurricular involvement (Wood et al., 2017). For the outcomes variables, the predictive of IAFREE should be tested towards rates of unemployment in the future (Marchbanks et al., 2014), levels of stress, mental disorders, decreased quality of life, illiteracy, and rates of crime and poverty (Dever and Raines, 2013; Nelson et al., 2016). In addition, future research could investigate these correlates through a longitudinal design (Tabuchi et al., 2018).

Finally, as previously stated, accomplishing the aforementioned research agenda might contribute to educational intervention programs. In line with Wood et al. (2017), it is possible to deduce a relationship between the five-factor of risk for school dropout (i.e., IAFREE) and the model called “Response to Intervention” (RTI). This method implies early intervention for students at risk for school failure (Fuchs and Fuchs, 2006). As RTI consists of a multi-tiered intervention model, for example, the dimensions of Student-Family and Student-Community of IAFREE could be useful to sustain in some contexts a “universal tier” intervention allowing encouraging parent- and community involvement. Otherwise, the Student–Student dimension (e.g., affective, psychological, and cognitive elements) matches the “indicated tier” related to intensive, multifaceted, and individualized interventions (Fuchs and Fuchs, 2006). In short, available future studies will allow testing of these hypotheses by addressing concerns and issues culturally relevant to each context (Nastasi et al., 2004).

## Data availability statement

The datasets for this article are not publicly available due to concerns regarding participant/patient anonymity. Requests to access the datasets should be directed to the corresponding author.

## Ethics statement

The studies involving human participants were reviewed and approved by Comité de Ética da Pesquisa Universidade Federal de Alagoas. Written informed consent to participate in this study was provided by the participants’ legal guardian or next of kin.

## Author contributions

All authors listed have made a substantial, direct, and intellectual contribution to the work, and approved it for publication.

## Funding

This work was supported by grants from the Ministério da Educação, Brazil (TED10974). The funders had no role in the study design, data collection and analyses, the decision to publish, or the preparation of the manuscript. Additionally, the funders had no influence on the interpretation of data and the final conclusions drawn.

## Conflict of interest

The authors declare that the research was conducted in the absence of any commercial or financial relationships that could be construed as a potential conflict of interest.

## Publisher’s note

All claims expressed in this article are solely those of the authors and do not necessarily represent those of their affiliated organizations, or those of the publisher, the editors and the reviewers. Any product that may be evaluated in this article, or claim that may be made by its manufacturer, is not guaranteed or endorsed by the publisher.
