# Supplementary material for: Advancing school dropout early warning systems: the IAFREE relational model for identifying at-risk students
Source: Front Psychol. 2023 Jul 31;14:1189283. doi: 10.3389/fpsyg.2023.1189283 (PMC10425558; doi:10.3389/fpsyg.2023.1189283)
Supplement: Supplementary file 1 [file Data_Sheet_1.docx]

***Supplementary Material***

Advancing School Dropout Early Warning System: The IAFREE Relational Model for Identifying At-Risk Students

# Supplementary Figures and Tables

*Table 1.* Reliability indices for IAFREE-36: dimensions and risk factors

| Risk dimensions for SD | Risk factors | Items | ⍺ | ω | C.R |
| --- | --- | --- | --- | --- | --- |
| Student-School | School materials | S3, S4, S23 | .53 | .53 | .54 |
|  | Student materials | S9, PA2, PA8 | .35 | .24 | .47 |
|  | Dimension S-School. | - | .55 | .56 | .56 |
| Student-School Professionals | Pedagogical inflexibility | S13, S18, S22 | .62 | .62 | .63 |
|  | Pedagogical quality | S12, S19, S20 | .59 | .60 | .60 |
|  | Dimension S-School P. | - | .76 | .76 | .76 |
| Student-Family | Family support | S16,PA7,PA11 | .32 | .24 | .35 |
|  | Pregnancy, parenting, and household care activities | S15, S21,PA12 | .32 | .38 | .38 |
|  | Dimension S-Fam. | - | .46 | .46 | .49 |
| Student-Community | Socio-educational measures and contexts of violence | PA3, PA5,PA10 | .66 | .67 | .66 |
|  | Accessibility, and school attendance | S8, S11, S14 | .62 | .61 | .62 |
|  | School-community distancing | S7, S17, PA1 | .36 | .38 | .40 |
|  | Dimension S-Comm. | - | .66 | .65 | .67 |
| Student-Student | Meaning of graduation/student engagement | S5, S6, S10 | .65 | .65 | .66 |
|  | Emotional and affective aspects/socioemotional health | S1, S2, S24 | .59 | .58 | .61 |
|  | Failures and age-grade distortion | PA4,PA6, PA9 | .74 | .77 | .76 |
|  | Dimension S-Stud. | - | .71 | .67 | .71 |
| IAFREE-36 | Overall reliability | 36 items | .85 | .87 | .89 |

*Note*. α = Cronbach’s alpha; ω = McDonald’s ômega; C.R = Composite Reliability statistic; S = Student answer; PA = Answer registered by school professional.

*Table 2.* Discrimination, and threshold parameters (b_1_–b_6_ = threshold) of the IAFREE-36

|  |  | Items | α | b_1_ | b_2_ | b_3_ | b_4_ | b_5_ | b_6_ | b_x_ |
| --- | --- | --- | --- | --- | --- | --- | --- | --- | --- | --- |
|  | SSc1 | S3 | 2.264 | 1.002 | 1.222 | 1.445 | 1.756 | 1.890 | 1.957 | 1.545 |
| SSc |  | S4 | 1.784 | 1.290 | 1.561 | 1.790 | 2.163 | 2.325 | 2.448 | 1.930 |
|  |  | S23 | 1.557 | 1.013 | 1.260 | 1.451 | 1.746 | 1.897 | 2.003 | 1.562 |
|  | SSc2 | S9 | .815 | 2.404 | 2.953 | 3.480 | 4.265 | 4.600 | 4.845 | 3.758 |
|  |  | PA2 | 1.640 | 1.563 | 1.912 | 2.126 | 2.211 | 2.376 | 2.467 | 2.109 |
|  |  | S8 | 6.960 | 1.374 | 1.682 | 1.910 | 2.071 | 2.200 | 2.293 | 1.922 |
|  | SP1 | S13 | 1.992 | 1.056 | 1.334 | 1.524 | 1.815 | 1.957 | 2.056 | 1.624 |
| SP |  | S18 | 2.776 | 1.198 | 1.472 | 1.685 | 2.044 | 2.180 | 2.274 | 1.809 |
|  |  | S22 | 2.565 | 1.072 | 1.317 | 1.491 | 1.730 | 1.841 | 1.936 | 1.565 |
|  | SP2 | S12 | 2.355 | 1.401 | 1.666 | 1.926 | 2.225 | 2.375 | 2.467 | 2.010 |
|  |  | S19 | 2.507 | 1.138 | 1.383 | 1.592 | 1.912 | 2.050 | 2.155 | 1.705 |
|  |  | S20 | 1.900 | .929 | 1.209 | 1.407 | 1.765 | 1.934 | 2.050 | 1.549 |
| SF | SF1 | S16 | .867 | 2.667 | 3.174 | 3.600 | 4.109 | 4.325 | 4.508 | 3.731 |
|  |  | PA7 | .944 | -.086 | .062 | .190 | 1.981 | 2.206 | 2.387 | 1.123 |
|  |  | PA11 | 2.283 | 1.157 | 1.386 | 1.641 | 1.834 | 2.069 | 2.269 | 1.726 |
|  | SF2 | S15 | 1.093 | .760 | 1.013 | 1.187 | 1.407 | 1.567 | 1.695 | 1.272 |
|  |  | PA12 | .895 | 3.579 | 3.942 | 4.477 | 6.351 | 6.520 | 6.936 | 5.301 |
|  |  | S21 | 3.331 | 1.104 | 1.315 | 1.481 | 1.717 | 1.816 | 1.890 | 1.554 |
|  | SC1 | PA3 | 4.412 | .420 | .653 | .826 | .966 | 1.229 | 1.441 | 0.923 |
| SC |  | PA5 | 4.036 | .342 | .585 | .759 | .878 | 1.172 | 1.387 | 0.854 |
|  |  | PA10 | 1.118 | 1.658 | 1.989 | 2.372 | 2.672 | 3.045 | 3.373 | 2.518 |
|  | SC2 | S8 | 2.431 | 1.321 | 1.597 | 1.857 | 2.304 | 2.438 | 2.560 | 2.013 |
|  |  | S11 | 3.074 | 1.383 | 1.643 | 1.846 | 2.193 | 2.286 | 2.347 | 1.950 |
|  |  | S14 | 2.062 | 1.426 | 1.705 | 1.930 | 2.256 | 2.392 | 2.483 | 2.032 |
|  | SC3 | S7 | 1.786 | 1.369 | 1.649 | 1.864 | 2.204 | 2.331 | 2.437 | 1.976 |
|  |  | S17 | 2.569 | 1.416 | 1.710 | 1.941 | 2.190 | 2.291 | 2.367 | 1.986 |
|  |  | PA1 | .690 | 2.099 | 2.869 | 3.572 | 3.857 | 4.470 | 5.007 | 3.646 |
|  | SSt1 | S5 | 2.488 | 1.197 | 1.460 | 1.676 | 2.039 | 2.207 | 2.302 | 1.814 |
| SSt |  | S6 | 2.939 | 1.138 | 1.394 | 1.599 | 1.972 | 2.094 | 2.215 | 1.735 |
|  |  | S10 | 2.244 | 1.085 | 1.322 | 1.513 | 1.849 | 1.983 | 2.087 | 1.640 |
|  | SSt2 | S1 | 2.479 | 1.013 | 1.199 | 1.367 | 1.668 | 1.821 | 1.910 | 1.496 |
|  |  | S2 | 2.450 | 1.004 | 1.289 | 1.474 | 1.762 | 1.899 | 2.008 | 1.573 |
|  |  | S24 | 1.397 | 1.237 | 1.520 | 1.731 | 2.053 | 2.233 | 2.372 | 1.858 |
|  | SSt3 | PA4 | 4.209 | .420 | .655 | .829 | .970 | 1.237 | 1.453 | 0.927 |
|  |  | PA6 | 4.199 | .339 | .580 | .752 | .871 | 1.164 | 1.379 | 0.848 |
|  |  | PA9 | 1.414 | 1.374 | 1.616 | 1.778 | 1.874 | 2.064 | 2.220 | 1.821 |

*Note.* a = discrimination parameter; b_1_–b_6_ = threshold; b_x_ = difficulty average value; S = Student answer; PA = Answer registered by school professional.

## Supplementary Figures

There are no supplementary figures.
